# Clinical, functional and radiological outcome after osteosynthesis of ankle fractures using a specific provocation test

**DOI:** 10.1186/s13018-024-04820-x

**Published:** 2024-06-02

**Authors:** Julian Zimmermann, Liv Zingg, Walter O. Frey, Michel Schläppi, Arby Babians, Urs Zingg

**Affiliations:** 1 Department of Orthopaedics and Traumatology, Limmattal Hospital, Urdorferstrasse 100, Zurich-Schlieren, 8952 Switzerland; 2 Movemed, Department of Sport Medicine, University Hospital Balgrist, Zurich, 8008 Switzerland; 3 Department of Orthopaedics and Traumatology, Hospital of Winterthur, Brauerstrasse 15, Postfach, Winterthur, 8401 Switzerland; 4 Klinik Hirslanden Zurich, Witellikerstrasse 40, Zürich, 8032 Switzerland

**Keywords:** Ankle fractures, Functional outcome, ORIF, Postoperative sporting activity, Provocation tests, Radiological outcome

## Abstract

**Background:**

Ankle fractures are frequent, and despite numerous publications on their treatment and outcome, there is a lack of precise data on the functional results in young, healthy and physically active patients. We hypothesized that patients who underwent open reduction and internal fixation (ORIF) for simple ankle fractures would have similar function compared to a healthy control group, whereas patients with complex fractures will have significant functional deficits. Furthermore, we postulate that there is a discrepancy between the radiological and the functional outcomes.

**Methods:**

A set of specific provocation tests was developed to evaluate the postoperative possibility of weight bearing, stop-and-go activities and range of motion. In combination with three questionnaires and a radiographic evaluation, the true functional outcome and the possibility of participating in sporting activities were investigated and compared with those of an age- and sex-matched control group.

**Results:**

A significant impairment was found in unilateral and simple ankle fractures. This impairment increased in tests including stop-and-go activities in combination with load bearing and with the complexity of the fractures. Concerning the subjective outcome, there was a significant adverse effect for daily activities without any difference in preoperative or postoperative sporting activity between the groups. No difference was found in the radiological assessment.

**Conclusions:**

Both simple and complex ankle fractures treated with ORIF have a significant and long-lasting impact on functional outcome in young and active patients. The radiological result is not associated with a good functional outcome.

**Trial registration:**

BASEC-Nr. 2018 − 01124.

**Supplementary Information:**

The online version contains supplementary material available at 10.1186/s13018-024-04820-x.

## Introduction

Ankle fractures are a common occurence with an incidence of approximately 168/100,000/year [1]. Sixty to 70% are unimalleolar, 15 to 20% are bimalleolar, and 7 to 12% are trimalleolar fractures (bimalleolar in combination with the Volkmann fracture) [2]. The typical patients are elderly females with low-energy distortion traumas or young males following sport accidents [1]. To describe the fracture pattern, a number of classification systems have been established, of which the Danis-Weber and the Lauge-Hansen systems are most commonly used [2, 3]. 

Unstable fractures, those with loss of joint congruence, such as Weber type B or C fractures, and bimalleolar fractures with and without combination with the Volkmann fracture are usually treated operatively. In contrast to pilon fractures, which include the distal plafond of the tibia and mostly need primary fixation with an external fixator prior to definitive surgery, malleolar fractures may be treated with primary definitive surgery or after initial temporary stabilization in a cast or with an external fixator, respectively. The treatment decision is mainly made by the soft tissue status or the trauma mechanism, whereas outcome is known to be inversely proportional to the fracture comminutions in pilon and malleolar fractures [4]. 

The high incidence of ankle fractures led to a great number of studies evaluating the operative techniques and their outcomes. The quality of these studies is often moderate. The cohorts are heterogeneous, the selection is biased, the numbers are low, and the outcome analyses usually include only questionnaires or simple movement tests. There is a need for more precise outcome data with more stringent study designs and ankle movement-focused diagnostic tests [5]. 

To our knowledge, there are no specific provocation tests that analyze the possible extent of movement, weight bearing and activity after operative treatment for ankle fractures. Especially for active patients, there is a lack of knowledge concerning the outcomes of their postoperative abilities in various physically strenuous activities. Therefore, specific ankle provocation tests were developed for this study and combined with a focused questionnaire on function and quality of life for physically active patients sustaining an ankle fracture and undergoing open reduction and internal fixation (ORIF).

We hypothesized that patients who underwent ORIF for simple ankle fractures would have similar functions compared to patients in the healthy control group, whereas patients with complex fractures would have significant functional deficits. Furthermore, we postulate that there is a discrepancy between the radiological and functional outcomes.

## Materials and methods

All patients aged between 18 and 51 years who underwent ORIF for simple or complex ankle fractures between 12/2012 and 03/2022 at our institution were assessed for eligibility. The simple fracture group consisted of patients with fractures of the lateral malleolus at the level of the syndesmosis (Weber B type). The complex fracture group consisted of patients with bi- or trimalleolar fractures and solitary Weber C-type fractures because of the more complex trauma mechanism involved compared to Weber B-type fractures. The two surgical groups were compared to an age- and sex-matched cohort of healthy volunteers without any history of pathology of the lower limb.

The exclusion criteria in the operative groups were any pathology of the lower limb, such as vascular disease, neurological disease, arthritis of any lower limb joint, pregnancy or proven infection of the osteosynthesis material. The minimum follow-up time was 24 months for patients who did not undergo hardware removal. The minimum follow-up for those who underwent hardware removal (usually 12 months after ORIF) was 12 months after hardware removal. The decision to remove the hardware was based on patient wishes after confirming complete radiological consolidation of the fractures. All patients and volunteers signed an informed consent form, and the study was approved by the State Ethical Committee.

### Open reduction and internal fixation (ORIF)

The technique of ORIF has been described and analyzed in several randomized trials and is the standard of care [3, 6]. 

Briefly, after incisions at the level of the fracture and visualization of the fragments, the fracture was anatomically reduced. If a Volkmann fracture was present and more than 25% of the joint surface was affected, as measured on plain lateral radiographs, and/or in displaced fracture situations of the posterior malleolar fragment, a fixation was deemed necessary [7–9, 9]. Internal fixation was performed with compression screws and plates (one-third, one-fourth tubular plate® or 2.7/3.5 mm LCP distal fibular plate®, DePuy Synthes) . The indication for a set screw depended on the fracture level and testing of the syndesmosis with an external rotation and lateral pull test (hook test) [10–12]. 

Intraoperative anterior-posterior and lateral X-rays were performed to check for joint congruence and quality of the reduction.

The wound was closed with subcutaneous sutures (Vicryl® 3 − 0, Ethicon) and non-resorbable skin sutures (Ethilon® 3 − 0, Ethicon). Postoperative treatment consisted of either partial or full weight bearing as well as the need for postoperative immobilization in a walker boot or cast depending on compliance, bone quality, complexity of the fracture and the need for a set screw.

### Functional outcome, ankle provocation tests and radiological assessment

Functional outcome was measured by a composite ankle provocation test and three questionnaires. This specific test was developed in cooperation with the University Centre for Prevention and Sports Medicine (Appendix 1). The test included the five following tests: dorsal flexion with weight bearing, Y-balance test, modified drop jump test, modified leg press test and maximum distance floor-to-heel test.

The range of motion of the ankle joint and the circumference of the thigh and the lower leg were analyzed. All tests were performed on both ankles. The range of motion (ROM) was measured, and the difference between the operated and non-operated ankles was used for comparison within the groups. In addition, pain was assessed with the visual analog scale (VAS) score, which ranges from 1 to 10 (maximum).

The three questionnaires consisted of the Foot and Ankle Ability Measure (FAAM) for function [13–15], the SF-36 questionnaire for measurement of quality of life and a homemade questionnaire (Appendix 2) to assess the frequency of sporting activity pre- and postoperatively.

The radiological examination consisted of weight-bearing anterior-posterior (mortise view) and lateral X-rays of both ankles, i.e., the operated and the contralateral ones. Medial and lateral clear spaces of the tibio-talar joint were defined as 4 or more millimeters, and the tibio-fibular clear space was defined as 5 or less millimeters on mortise views in healthy, uninjured, weight-bearing ankles. Measurements were performed from the lateral border of the medial malleolus and the medial border of the talar dome 5 mm inferior to the superior talar joint line for the medial clear space. The distance between the medial border of the lateral malleolus and the lateral border of the talar dome 5 mm inferior to the superior talar joint line was used for the lateral clear space. The tibio-fibular clear space was measured from the point 1 cm above the lateral edge of the inferior tibial joint line (anterior ridge of the tibia) to the corresponding horizontal point at the fibula. Medial and lateral clear spaces smaller than 4 mm and tibio-fibular clear spaces larger than 5 mm were considered pathological [16, 17]. 

Furthermore, X-rays were analyzed for signs of arthritic changes, and tibio-talar joint congruence was examined via mortise and lateral views according to the van Dijk classification [18]. The X-rays were analyzed independently by a senior trauma surgeon and a radiologist specializing in musculoskeletal radiology to rule out anatomic pathologies, arthritis, and failure of the osteosynthesis material. The control group was not investigated radiologically for ethical reasons (radiation exposure).

### Statistical analysis

The data were coded anonymously and collected using Microsoft Excel 2013®, version 15.0 (Microsoft Corp., Redmond, Washington). All the statistical analyses were performed by an independent professional statistician, and a power analysis was performed. To detect an effect size of 0.6 with an alpha level of 0.05 and a power level of 0.80 for the Kruskal‒Wallis test, a total sample size of 90 patients (30 patients per group) was calculated.

Statistical analysis was performed using R statistical software (Windows version 4.2.1; R Foundation for Statistical Computing). The data were checked for normality and homogeneity of variance using the Shapiro‒Wilk and Levene tests, respectively. For each group, the difference between the injured and the non-injured leg was calculated, and this delta was used for comparisons between the groups. One-way analysis of variance (ANOVA) followed by post hoc tests with Bonferroni correction was used for multiple comparisons. Categorical variables were analyzed by the chi-square test or Fisher’s exact test, as appropriate. Nonparametric data were compared by the Kruskal‒Wallis test. A *p* value of less than 0.05 was considered to indicate statistical significance.

## Results

The baseline characteristics and demographic data of the patients are summarized in Table 1. Overall, between 2012 and 2022, 570 patients of all ages were treated operatively for an ankle fracture. Of these, 30 patients with simple and 26 in the complex fractures, respectively, met all the inclusion criteria and consented to the participation. These groups were matched with a control of 38 participants. The mean follow-up was 13 months in both groups. There was no significant difference in age or sex between the groups. There was a significant difference in BMI between the control and the operative patients.

Hardware removal occurred significantly more often in the complex group. Nicotine consumption was significantly more frequent in the operative groups.

**Table 1:** Demographic data of the groups

	Control	Simple	Complex	*P* values		
	*n* = 38	*n* = 30	*n* = 26	control - simple	control - complex	simple - complex
Mean Age [y] (SD)	35.9 (10.5)	35.3 (9.2)	36.1 (10.3)	0.966	0.995	0.946
Nicotine consumption (%)	6 (16)	12 (40)	11 (42)	0.049	0.038	1.000
Numbers of hardware removal (%)	-	20 (67)	24 (92)	-	-	0.025
Numbers of males (%)	18 (47)	10 (34)	11 (42)	0.418	0.886	0.750
Mean BMI [kg/m^2^] (SD)	23.0 (2.6)	27.4 (4.6)	25.6 (3.8)	< 0.001	0.018	0.186
Type of injury				-	-	0.310
Sport (%)	-	14 (50)	17 (65)			
Home (%)	-	12 (43)	6 (23)			
Bike/Car (%)	-	2 (7)	3 (12)			

### Clinical outcome and ankle provocation tests

Weight bearing with bodyweight was tolerated throughout all groups, and both legs were not restricted.

All patients had good functioning ROMs with no passive or active impairment in plantar flexion. The range of active dorsal extension (without weight bearing) was greater in the simple and complex groups than in the control group. Compared to that on the uninjured side, the active dorsal extension (without weight bearing) was lower on the injured side. No difference was detected between the simple and complex groups.

Table 2 shows the results of the ankle provocation tests.

**Table 2:** Results of clinical outcome and provocation tests (range). Differences between groups are shown in Table 3

	Control		Simple		Complex	
	*n* = 38		*n* = 30		*n* = 26	
	right	left	injured	uninjured	injured	uninjured
Active plantar flexion [°]	50 (14–72)	50 (20–70)	41 (21–62)	40 (23–68)	29 (19–45)	30 (15–60)
Active dorsal extension [°]	13 (2–60)	12 (2–60)	17 (4–45)	18 (2–45)	30 (12–50)	36 (15–50)
Forced dorsal extension [°]	13 (4–21)	12 (4–19)	10 (4–15)	12 (5–16)	7 (0–17)	12 (7–18)
Y Balance Test total [cm]	109 (86–142)	111 (87–159)	107 (69–150)	107 (65–159)	99 (63–142)	101 (63–164)
- anterior [cm]	100 (75–135)	98 (80–145)	90 (50–110)	90 (40–115)	80 (50–130)	69 (40–125)
- posteromedial [cm]	108 (80–135)	105 (75–150)	100 (72–135)	100 (73–125)	83 (50–150)	95 (50–130)
- posterolateral [cm]	98 (80–140)	100 (80–150)	95 (70–115)	100 (10–125)	80 (50–135)	93 (50–130)
Drop-jump test [cm]	115 (55–180)	110 (55–200)	105 (0-195)	115 (25–180)	88 (0-180)	106 (20–185)
Floor-to-heel distance [cm]	10 (6–13)	10 (6–13)	9 (5–13)	9 (5–13)	9 (3–13)	10 (6–15)
Leg-press [kg]	63 (27–135)	63 (27–144)	63 (35–117)	72 (27–130)	75 (20–120)	85 (30–140)
Circumference [cm]						
- Thigh [cm]	46 (37–51)	46 (37–52)	49 (41–60)	49 (40–60)	47 (37–55)	49 (38–60)
- Lower leg [cm]	37 (31–42)	37 (31–42)	38 (34–45)	38 (34–63)	38 (32–42)	38 (33–51)

The differences between the groups are shown in Table 3. A negative value corresponds to a deficit in the ORIF side compared to the non-operated side. The simple fracture group showed significantly worse outcomes in all tests, except for the floor-to-heel test. Patients with complex fractures had significantly worse functional outcomes in all the provocation tests compared to the controls. Compared to those in the simple fracture group, the forced dorsal extension and floor-to-heel test results were significantly worse after complex fractures.

Patients with ankle fractures had greater circumferences of both the thigh and the lower leg on both sides, whereas significance was found only for the lower leg.

**Table 3:** Median relative difference in % (range) between the operated leg and the uninjured leg in the provocation tests. A negative value corresponds to a deficit of the operated side in comparison to the uninjured leg

	Control (difference in %)	Simple (difference in %)	Complex (difference in %)	*P* value Control - Simple	*P* value Control - Complex	*P* value Simple - Complex
Active plantar flexion [°]	0 (-33-73)	0 (-28-43)	0 (-50-50)	1.000	1.000	0.460
Active dorsal extension [°]	13.3 (-88-400)	-11 (-56-125)	0 (-60-50)	1.000	0.073	0.113
Forced dorsal extension [°]	0 (-33-75)	‘-13 (-40-15)	-47 (-100-0)	< 0.001	< 0.001	< 0.001
Drop-jump test [cm]	0 (-14-47)	‘-5 (-100-220)	-10 (-100-5)	0.020	< 0.001	0.110
Floor-to-heel distance [cm]	0 (-13-29)	0 (-36-40)	-11 (-50-25)	1.000	< 0.001	0.008
Leg-press [kg]	0 (-13-30)	-7 (-30-78)	-12 (57 − 20)	0.005	< 0.001	0.402
Circumference thigh [cm]	0 (0.5 − 4)	0 (-8-28)	0 (-10-6)	1.000	0.860	0.510
Circumference lower leg [cm]	0 (-3-8)	0 (-29-6)	0 (-22-6)	0.035	0.001	0.717

### Subjective outcome

The patients with simple and complex fractures showed significant impairments in daily activities in comparison to the controls, as shown in Table 4. In the SF-36, all patients had significant limitations in physical and social functioning and pain compared to the healthy volunteers, but there was no difference in these limitations between the two operatively treated groups.

There was no significant difference in preoperative or postoperative sporting activity between the groups. The simple group showed a slight decrease in physical activity postoperatively (83% vs. 73%), which was not significant (*p* = 0.894). The complex group showed the same physical activity levels pre- and postoperatively (77%). 60% of the simple fracture group and 50% of the complex fracture group could participate in stop-and-go activities with or without body contact (Table 5).

**Table 4:** Subjective outcomes according to the Foot and Ankle Ability Measure (FAAM) and quality-of-life questionnaire (SF-36) scores

	Control (*n* = 38)	Simple (*n* = 30)	Complex (*n* = 26)	*P* value Control - Simple	*P* value Control - Complex	*P* value Simple - Complex
FAAM	1 (0.911-1.000)	0.948 (0.482-1.000)	0.896 (0.384-1.000)	0.001	< 0.001	0.957
SF-36 total score	90 (74–100)	86 (48–100)	87 (35–97)	0.323	0.021	1.000
Energy/fatigue	70 (25–100)	65 (20–100)	65 (25–80)	0.199	0.180	1.000
Physical funtioning	100 (75–100)	98 (40–100)	95 (30–100)	0.005	< 0.001	1.000
Pain	100 (58–100)	100 (23–100)	90 (33–100)	0.005	< 0.001	0.630
General health	75 (55–100)	75 (30–100)	78 (30–100)	1.000	0.988	1.000
Role limitations due to physical health	100 (100–100)	100 (25–100)	100 (0-100)	0.127	0.042	1.000
Role limitations due to emotional problems	100 (67–100)	100 (67–100)	100 (0-100)	0.359	0.245	1.000
Social functioning	100 (50–100)	100 (50–100)	94 (38–100)	0.090	0.023	1.000
Emotional well-being	80 (56–100)	84 (44–100)	80 (28–96)	1.000	0.911	0.830

**Table 5:** Results of pre- and postoperative sport activities

	Control (*n* = 38)	Simple (*n* = 30)	Complex (*n* = 26)	*P* value Control - Simple	*P* value Control - Complex	*P* value Simple - Complex
Preoperative active in sports (%)	32 (84)	25 (83)	20 (77)	1.000	0.684	0.588
Postoperative active in sports (%)	-	22 (73)	20 (77)	0.092	0.288	1.000
Stop and go sports with body contact (%)	11 (29)	10 (33)	6 (23)	0.975	0.904	0.727
Stop and go sports without body contact (%)	8 (21)	8 (27)	7 (27)	0.884	0.892	1.000
Endurance sports like cycling or swimming (%)	10 (26)	4 (13)	6 (23)	0.377	1.000	0.505
Sports and activities without load for the upper ankle (%)	3 (8)	0	1 (4)	0.258	1.000	0.474
No answer (%)	6 (16)	8 (27)	6 (23)	0.551	0.776	1.000
Postoperative more active in sports (%)	-	6 (20)	2 (8)	-	-	0.449

### Radiological assessment

There was no significant difference in the radiological assessment points between the uninjured and injured ankles in either group. Most patients in this study had a medial or lateral clear space of less than 4 millimeters on the mortise view on both ankles. Except for narrowing of the joint space, no signs of arthritic change were detected. A summary of the radiological findings is shown in Table 6.

**Table 6:** Results of the radiological assessment of weight-bearing mortise views of both ankles

	Simple (*n* = 30)		*P* value	Complex (*n* = 26)		*P* value
	injured	uninjured		injured	uninjured	
Medial tibio-talar clear space < 4 mm (%)	29 (97)	30 (100)	1.000	25 (96)	26 (100)	1.000
Lateral tibio-talar clear space < 4 mm (%)	30 (100)	30 (100)	1.000	26 (100)	26 (100)	1.000
Tibio-fibular clear space > 5 mm (%)	12 (40)	9 (30)	0.757	2 (8)	2 (8)	1.000

## Discussion

The major finding of this study is the significant impairment of the true functional outcome after ORIF for both simple and complex ankle fractures in young and physically active patients, compared to healthy volunteers. These findings occurred despite proven anatomical reduction and fixation, and no arthritic findings in the radiological assessment.

Contrary to our hypothesis of a similar functional outcome in operatively treated simple ankle fractures, a clear impairment in forced dorsal extension was found in this group compared to the control.

The impairment in forced dorsal extension after ORIF may be caused by the following mechanisms: an altered length‒tension relationship with changed neuromuscular mechanisms; scarring of the joint capsule due to the surgical approach; concomitant soft tissue and ligamentous (deltoid ligament) injury; and/or postoperative immobilization in a cast or walker boot that may cause shortening of the gastroc-soleus complex [19]. Furthermore, the duration of postoperative immobilization seems to have an impact on the severity of the impairment of range of motion [20–22]. The active and forced dorsal extension was more limited in the complex group. Complex ankle fractures require a more strict and often longer immobilization than simple ankle fractures that may be treated with early weight bearing and physiotherapy-supported movement exercises [21, 23, 24]. Furthermore, complex fractures, including ankle fracture dislocations, are more often treated in two steps. First, the closed reduction and temporary external fixation followed by the ORIF. This may be another cause of impaired postoperative ROM [9, 25–27]. Unfortunately, this impairment in ROM does not decrease even after several years [20]. 

Interestingly, the ROM of active dorsal extension of the ankle without weight bearing was greater in both operatively treated groups compared to the control group. For this finding we have no scientific explanation, although it could be that the postoperative physiotherapy led to increased ROM as a side effect.

The literature has primarily focused on the timing and amount of weight bearing and immobilization after ORIF for ankle fractures. There is a lack of precise information on the functional outcomes [23, 28, 29]. To our knowledge, studies investigating functional outcomes have not examined patients themselves but rather obtained results indirectly through questionnaires [29, 30]. 

In weight-bearing exercises, such as the drop-and-jump test, the operated leg did not achieve the same results as the uninjured leg in either postoperative group. Patients who had complex fractures had significantly worse outcomes. This finding might be caused by an initial deltoid ligament injury (in patients without a fracture of the medial malleolus), which results in a more pronounced feeling of ankle instability and the resulting fear of reinjuring the ankle, despite the absence of clinical or radiological signs of instability. Importantly, pain was not the limiting factor. However, in the leg press test, the complex fracture group surprisingly had the best outcome. This might be caused by a more intense and longer period of postoperative physiotherapy-supported rehabilitation. This is supported by the finding that the operative patients had a larger circumference of the thigh and the lower leg. Obviously, we do not know whether this difference already existed preoperatively, and we did not find any published studies investigating forced weight bearing or its correlation with circumferences of the lower leg. The complex group showed worse results in the drop jump test but better results in the leg press test than the simple group. One reason for this finding might be the nature of these specific tests. The drop jump requires explosive power and maximum ankle stability, whereas the leg press test creates a more static load situation for the ankle joint (open versus closed chain exercises). Complex fractures can lead to a chronic posttraumatic ankle instability. This causes pain and uncertainty with stop-and-go exercises and might lead to chronic recurring ankle sprains or even fractures. Beside intense physiotherapy-supported rehabilitation, conservative treatments like ankle supporters and kinesio- taping have been shown to improve feelings of instability and gait dysfunction, respectively [31]. 

Concerning the subjective outcome, both groups showed an impairment in the quality of life with a focus on physical and social functioning as well as some limitations in daily living, while pain did not play a dominant role. These findings are consistent with the literature, and impairment might even exist two years after surgery awhich may be associated with increasing age [32–37]. The control group did not reach the maximum values in the questionaires, which perhaps indicates a psycho-social or other distorting influence [38]. 

Despite proven impairments in range of motion, functional testing and quality of life, most patients were able to participate in postoperative strenuous physical activities. The literature concerning this topic is not only contradictory but also scarce and heterogeneous. Some authors have described severe impairment in postoperative strenuous physical activities in simple ankle fractures, while others have shown that almost all patients return to preoperative sports and activity levels, even in athletes [35, 39–44]. Factors influencing the return to sports are age, talar osteochondral lesions, syndesmotic injury, preoperative activity level and motivation, especially in professional athletes [45, 46]. In addition, the complexity of ankle fractures plays an important role. Complex fractures are more frequently combined with ankle dislocations, another risk factor for persistent ankle instability and poor outcomes [46–49]. 

We hypothesized that there would be a discrepancy between the radiological outcome and the clinical and/or functional outcome. This was confirmed as all the X-rays showed an anatomical reduction and a fully healed fracture. Most patients, including those in the control group, had medial and lateral clear spaces symmetrically smaller than 4 mm, which might be one radiological sign of developing arthritis according to van Dijk and Scranton [18, 50–52]. However, no other signs of arthritic changes, such as osteophyte formation, subchondral sclerosis or subchondral cysts, were observed. One reason for this finding may be the mean follow up time of 13 months (after hardware removal), as this is rather short for the development of osteoarthritis. In case of severe ankle and hindfoot osteoarthritis, several possibilities including different types of tibio-talar or tibio-talo-calcaneal arthrodesis (screw, plate, hindfoot nail) are possible [53]. 

The quality of measurements on plain ankle radiographs are still the subject of discussion because they could be influenced by a variety of factors, including the positioning of the foot and ankle, individual joint spaces depending on age, sex and height, and an inter-observer variability [52, 54, 55]. 

Concerning the syndesmosis, more patients with simple fractures had a tibio-fibular space over 5 mm compared to the patients in the complex group. In these latter fractures the syndesmosis was always treated by ligamentous and/or osseous stabilization. In contrast, in type Weber B fractures, this stabilization depended on the intraoperative clinical and radiological assessment (hook test, external rotation test). Traditional measurements on X-rays for syndesmotic injury do not correlate with findings on the more sensitive and specific MRIs, except for the tibiofibular overlap [52]. Furthermore, approximately 25% of patients suffering from a stage four supination-external rotation type fracture have a combination of the medial malleolar fracture with an injury to the deltoid ligament complex [10–12, 56]. These findings cast a general doubt on the accuracy of measuring joint spaces or the tibio-fibular clear space on plain radiographs to evaluate syndesmotic injury.

Several limitations warrant mentioning. First, this study was retrospective, and the surgeons were heterogeneous. However, all surgeons were trained in Switzerland by the AO principles, and thus, a ‘Unité de doctrine’ was present. Second, fractures were grouped only by radiological criteria, categorizing type Weber B fractures without accompanying fractures of the posterior or medial malleolus as a simple group. All other malleolar fractures were grouped as complex fractures. One could argue that Weber B fractures may have occult ligamentous injuries and that a more sophisticated grouping, e.g., MRI-based, would be more scientifically accurate . However, the great majority of surgeons group patients according to the Danis-Weber classification, therefore we used this widely popular classification system. In addition, the follow-up radiological evaluation was performed on plain a.p. and lateral weight bearing radiographs, whereas CT or MRI-based evaluation would have provided more specific information about osseous, cartilaginous and ligamentous status of the ankle.

Third, the number of patients was moderate due to the very strict inclusion and exclusion criteria. In particular, the maximum age of 51 years and the requirement of an absence of any pathology to the lower extremity led to the exclusion of many patients. Nevertheless, we believe that creating very homogenous groups leads to more precise results. Overall, it was difficult to motivate the patients to undergo the amount of testing, which also included radiological exams. Furthermore, we did not evaluate the sport and activity level by a well-established score such as Tegner activity level score. However, to our knowledge, no other study has assessed the outcome of a specific group of young and active patients with such specific function tests, and the literature lacks the necessary homogeneity and thus the true functional outcome. Despite the moderate number of patients, the study reaches enough statistical power to show the differences in the functional outcome of these frequent fractures. Additionally, we present several functional tests that can be used for further studies in this field.

## Conclusion

Simple as well as complex ankle fractures treated with ORIF have a significant and long-lasting impact on the functional outcome in young and active patients. A good radiological result does not indicate an equally good functional outcome. The results underline the importance of restoring ankle stability by addressing all ligamentous and osseous injuries, especially in high-demand patients.

### Electronic supplementary material

Below is the link to the electronic supplementary material.

Supplementary Material 1

Supplementary Material 2

Supplementary Material 3

Supplementary Material 4

## Data Availability

No datasets were generated or analysed during the current study.

## Abbreviations

ANOVA: One-way analysis of variance
AO: Arbeitsgemeinschaft Osteosynthese (german)
BMI: Body mass index
FAAM: Foot and Ankle Ability Measure
MRI: Magnetic resonance imaging
ORIF: Open reduction and internal fixation
ROM: Range of motion
