# Supplementary material for: Clinical, functional and radiological outcome after osteosynthesis of ankle fractures using a specific provocation test
Source: J Orthop Surg Res. 2024 Jun 2;19:327. doi: 10.1186/s13018-024-04820-x (PMC11145828; doi:10.1186/s13018-024-04820-x)
Supplement: Supplementary file 1 — Supplementary Material 1 [file 13018_2024_4820_MOESM1_ESM.docx]

**Appendix 2 – Questionnaires (english):**

## Case Report Form (CRF)

**CASE REPORT FORM - CONFIDENTIAL**

**Title:**

*Clinical, functional and radiological outcome after osteosynthesis of ankle fractures using a specific provocation test- a combined retro- and prospective study*

**Participant Number:** .................................................................................................................

**Date:**………………………………………………………………………………………..

**Inclusion criteria:**

- Age > 18 and < 51 years
- Open reduction and internal fixation (ORIF) of an ankle fracture
- Time from surgery to study investigation: min. 2 years
- Time to hardware removal (if done so): min. 1 year
- Time from hardware removal to study investigation: min. 1 year
- Signed Patie informed consent
- Patient is able to give informed consent

**Exclusion criteria:**

- Minors or persons not capable of judging
- Further pathologies of the lower extremities (further fracturs, Osteoarthritis, performed surgery)
- Preexisting vascular diseases or neuropathy of the affected or unaffected limb.
- Other medical conditions like pneumopathies, heart failure, osteoporosis, diabetes mellitus
- Infection of the wardware (osteosynthesis)
- Patient does not agree with the informed consent

**Patient data and characteristics:**

- Sex: ……………………………………………………………………............
- Date of birth: …………………………………………………………………...
- Height, Weight, BMI:…………………………………………………………………………
- Main leg: Left:  Right:
- Side of surgery: Left:  Right:
- Ankle Plantar Flexion/Dorsal Extension: …………………………..
- Nicotine consumption pre surgery:
- Nicotine consumption after surgery:
- Medication with NSAR (Irfen, Ibuprofen, Ecofenac, Diclofenac etc.) after surgery: Yes  No
- If yes, < 2 weeks: , 2-6 weeks: , > 6 weeks

**Injury characteristics and therapy:**

- Kind of accident: Traffic  Sports  Home
- Date of definitive surgery (ORIF): ………………………………………………………….
- Date of hardware removal: ………..………………………………………………...

**Questionnaires**

- Foot and Ankle Ability Measure (FAAM)
- SF 36 Questionnaire (Maximum 100 Points)
- Sports Questionnaire (self created)

**Diagnostics:**

- X Ray Ankle lateral and a.p.

Findings:

Existing hardware:

Osteoarthritis:

Further findings: ……….……………………………………………

- Test of Dorsal Extension with weight bearing:

Distance Wall – heel (cm) affected (surgery performed) ankle: ……….

Distance Wall – heel (cm) non-affected ankle: ……….

VAS Score after testing (0 = no pain – 10 = maximum pain):

- Y-Balance Test:

Does the patient know how to perform the test already?

Leg length affected limb (cm): ……….

Leg length non-affected limb (cm): ……….

Maximum distance to the heel of the affected limb (cm):

anterior ………posteromedial………posterolateral…………….

Maximum distance to the heel of the non - affected limb (cm):

anterior ………posteromedial………posterolateral…………….

VAS Score after testing (0 = no pain – 10 = maximum pain):

- Drop-Jump-Test:

Maximum distance of the affected limb (cm): ………………………………….

Maximum distance of the affected limb (cm): …….……………………………

VAS Score after testing (0 = no pain – 10 = maximum pain):

- Floor to heel Distance affected ankle (cm) in upright position (standing): ………………………………………..
- Floor to heel Distance non - affected ankle (cm) in upright position (standing): ………………………………………..
- Leg-Press-Test:

**With …… KG** weight at least 3 cm from the foot plate were reached

VAS Score after testing (0 = no pain – 10 = maximum pain):

- Circumference thigh affected limb (10 cm above superior pole of the patella, cm): ……
- Circumference lower leg affected limb (maximum circumference, cm): ….
- Circumference thigh non - affected limb (10 cm above superior pole of the patella, cm): ……
- Circumference lower leg non - affected limb (maximum circumference, cm): ….

## Pre- postoperative sporting activity questionnaire - Patient

Clinical, functional and radiological outcome after osteosynthesis of lateral ankle fractures using a specific provocation test- a combined retro- and prospective study

Sex: ☐ F ☐ M

Year of birth:

Participant number.:

**3. Questionnaire of sportive activity before and after ORIF of an ankle fracture**

**Have you been active in sports before you broke your ankle?**

If yes,

1x/week

2-3x/week

3-5x/week

Yes, every day

No:

**If yes, what kind of sports?**

………………………………………………………………………………………………………………………

**Have you been a competitive athlete before you broke your ankle?**

Yes:

No:

**Did your sportive activity change after finishing rehabilitation (6 months after surgery) ?**

Yes:

No:

If yes,

**after surgery different kind of sports:**

What kind of ………………………………………………………………………………………….

**After surgery more often than pre-surgery:**

**After surgery less often than pre-surgery:**

…if yes, now

1x/week

2-3x/week

3-5x/week
