# Supplementary material for: Clinical, functional and radiological outcome after osteosynthesis of ankle fractures using a specific provocation test
Source: J Orthop Surg Res. 2024 Jun 2;19:327. doi: 10.1186/s13018-024-04820-x (PMC11145828; doi:10.1186/s13018-024-04820-x)
Supplement: Supplementary file 2 — Supplementary Material 2 [file 13018_2024_4820_MOESM2_ESM.docx]

**Appendix 2 – Questionnaires (german):**

## Case Report Form (CRF)

**CASE REPORT FORM - VERTRAULICH WENN AUSGEFÜLLT**

**Studientitel:**

*Clinical, functional and radiological outcome after osteosynthesis of ankle fractures using a specific provocation test- a combined retro- and prospective study*

**Teilnehmer Nr:** .................................................................................................................

**Visitendatum:**………………………………………………………………………………………..

**Einschlusskriterien:**

- Alter über 18 und unter 51 Jahren
- Stattgehabte osteosynthetische Versorgung einer Sprunggelenksfraktur
- Zeitabstand Operation bis Nachkontrolle: mind. 2 Jahre
- Zeitabstand bei stattgehabter Osteosynthesematerialentfernung: mind 1 Jahr
- Zeitabstand bis zur Osteosynthesematerialentfernung: mind. 12 Monate
- Patient unterschreibt den informed consent nach erfolgter Aufklärung über die Studie
- Patient ist fähig informed consent zu geben

**Ausschlusskriterien:**

- Minderjährige oder nicht urteilsfähige Patienten
- Weitere Pathologien der unteren Extremitäten (weitere Frakturen, vorbestehende Athrose oder Operation der ipsi- und/oder kontralateralen Seite)
- Vorbestehende peripher arterielle Verschlusskrankheit (paVk) oder Neuropathie der betroffenen oder kontralateralen Seite
- Relevante internistische Begleiterkrankungen wie Pneumopathien, Herzinsuffizienz, Osteoporose und Diabetes mellitus
- Auftreten eines Osteosynthesematerial assoziierten Infektes
- Patient unterschreibt informed consent nicht

**Patientendaten und -charakteristika:**

- Geschlecht: ……………………………………………………………………............
- Geburtsdatum (Alter): …………………………………………………………………...
- Grösse, Gewicht, BMI:…………………………………………………………………………
- Standbein: Links:  Rechts:
- OP: Links:  Rechts:
- Plantarflexion/Dorsalextension im OSG (nach Neutral-Null-Methode): …………………………..
- Nikotinkonsum vor Operation:
- Nikotinkonsum nach Operation:
- NSAR (Irfen, Ibuprofen, Ecofenac, Diclofenac etc.) Einnahme nach Operation: Ja  Nein
- Wenn ja, < 2 Wochen: , 2-6 Wochen: , > 6 Wochen

**Krankheitscharakteristik / Therapie:**

- Ursprüngliche Unfallart: Verkehrsunfall  Sport  Zuhause
- Datum der osteosynthetischen Versorgung: ………………………………………………………….
- Datum der Osteosynthesematerialentfernung:

………..………………………………………………...

**Symptome bei Nachkontrolle gemäss „Foot and Ankle Ability Measure (FAAM), SF 36, Sportfragebogen“ Fragebogen**

**Fragebogen**

- Foot and Ankle Ability Measure (FAAM)
- SF 36 Fragebogen (Maximum 100 Punkte)
- Eigener Fragebogen

**Diagnostik:**

- Röntgen OSG lateral, a.p.

Befund:

Verbliebenes Osteosynthesematerial:

Arthrose:

Andere Befunde: ……….……………………………………………

- Test der Dorsalflexion unter Belastung:

Abstand Wand-Ferse (in cm) operiertes OSG: ……….

Abstand Wand-Ferse (in cm) kontralaterales OSG: ….

VAS Score nach Test (0 = kein Schmerz – 10 = nicht erträglicher Schmerz):

- Y-Balance Test:

Ist dem/der Patienten(in) der Test bereits bekannt?

Beinlänge operiertes OSG (in cm): ……….

Beinlänge kontralaterales OSG (in cm): ……….

Maximal erreichte Distanz bis Ferse (in cm) mit operiertem OSG :

anterior ………posteromedial………posterolateral…………….

Maximal erreichte Distanz bis Ferse (in cm) mit kontralateralem OSG:

anterior ………posteromedial………posterolateral…………….

VAS Score nach Test (0 = kein Schmerz – 10 = nicht erträglicher Schmerz): ……….

- Drop-Jump-Test:

Maximal erreichte Distanz (in cm) mit operiertem OSG: ………………………………….

Maximal erreichte Distanz (in cm) mit kontralateralem OSG: …………………………….

VAS Score nach Test (0 = kein Schmerz – 10 = nicht erträglicher Schmerz):

- Boden-Fersenabstand operiertes OSG (in cm) ohne Belastung im Stehen: ………………………………………..
- Boden-Fersenabstand kontralaterales OSG (in cm) ohne Belastung im Stehen: ………………………………………..
- Leg-Press-Test:

**Mit …… KG** Belastung wurden mindestens 3 cm erreicht

VAS Score nach Test (0 = kein Schmerz – 10 = nicht erträglicher Schmerz):

- Umfang Oberschenkel **operiertes Bein** (10 cm oberhalb des Patellaoberrandes, in cm): ……
- Umfang Unterschenkel **operiertes Bein** (Maximalumfang, in cm): ….
- Umfang Oberschenkel **kontralaterales Bein** (10 cm oberhalb des Patellaoberrandes, in cm): ...
- Umfang Unterschenkel kontralaterales Bein (Maximalumfang, in cm): ….

## Pre- postoperative sporting activity questionnaire - Patient

Clinical, functional and radiological outcome after osteosynthesis of lateral ankle fractures using a specific provocation test- a combined retro- and prospective study

Geschlecht: ☐ W ☐ M

Jahrgang:

Teilnehmer Nr.:

**3. Fragebogen zur sportlichen Betätigung vor und nach operativer Versorgung einer Sprunggelenksfraktur**

**Waren Sie vor Ihrem Sprunggelenksbruch resp. vor der operativen Versorgung desselben sportlich aktiv?**

Wenn ja,

1x/Woche

2-3x/Woche

3-5x/Woche

Täglich

Nein:

**Wenn ja, welche Sportart haben Sie mehrheitlich ausgeführt?**

………………………………………………………………………………………………………………………

**Waren Sie vor Ihrem Sprunggelenksbruch resp. vor der operativen Versorgung desselben Leistungssportler oder in einem Verein mit regelmässiger sportlicher Betätigung?**

Ja:

Nein:

**Haben sich Ihre sportliche Betätigung und deren Häufigkeit nach Abschluss der Rehabilitationsphase (sechs Monate nach Operation) verändert?**

Ja:

Nein:

Wenn ja,

**Nach der Operation andere sportliche Betätigung:**

Welche:…………………………………………………………………………………………………………….

**Nach der Operation häufiger sportliche Betätigung:**

**Nach der Operation seltenere sportliche Betätigung:**

…wenn ja, aktuell…

1x/Woche

2-3x/Woche

3-5x/Woche
