# Supplementary material for: Clinical, functional and radiological outcome after osteosynthesis of ankle fractures using a specific provocation test
Source: J Orthop Surg Res. 2024 Jun 2;19:327. doi: 10.1186/s13018-024-04820-x (PMC11145828; doi:10.1186/s13018-024-04820-x)
Supplement: Supplementary file 3 — Supplementary Material 3 [file 13018_2024_4820_MOESM3_ESM.docx]

**Clinical, functional and radiological outcome after osteosynthesis of ankle fractures using a specific provocation test**

Julian Zimmermann(1)*, Liv Zingg(1)*, Walter Frey (2), Michel Schläppi (3), Arby Babians (1) , Urs Zingg (1)

18.03.2024

*Contributed equally as first authors

1. Department of Surgery, Limmattal Hospital, Zurich-Schlieren, Switzerland

2. Movemed, Department of Sport Medicine, Balgrist, Zurich, Switzerland

3. Department of Orthopaedics and Traumatology, Hospital of Winterthur, Switzerland

Corresponding Author: J. Zimmermann, Limmattal Hospital, Urdorferstrasse 100, 8952 Zurich-Schlieren, Switzerland, E-Mail: jbf.zimmermann@gmail.com, Telephone: + 41 76 213 80 55

**Full author information:**

Liv Zingg, Limmattal Hospital, Urdorferstrasse 100, 8952 Zurich-Schlieren, Switzerland, E-Mail: liv.zingg@icloud.com

Walter Frey, Movemed, Department of Sport Medicine, Balgrist, Forchstrasse 319, 8008 Zurich, Switzerland, E-Mail: welcome.walterofrey@hin.ch

Michel Schläppi, Department of Orthopaedics and Traumatology, Hospital of Winterthur, Brauerstrasse 15, Postfach 8401 Winterthur Switzerland, E-Mail: michel.schlaeppi@ksw.ch

Arby Babians, Limmattal Hospital, Urdorferstrasse 100, 8952 Zurich-Schlieren, Switzerland, E-Mail: arby.babians@spital-limmattal.ch

Urs Zingg, Limmattal Hospital, Urdorferstrasse 100, 8952 Zurich-Schlieren, Switzerland, E-Mail: urs.zingg@spital-limmattal.ch
