# Supplementary material for: Clinical, functional and radiological outcome after osteosynthesis of ankle fractures using a specific provocation test
Source: J Orthop Surg Res. 2024 Jun 2;19:327. doi: 10.1186/s13018-024-04820-x (PMC11145828; doi:10.1186/s13018-024-04820-x)
Supplement: Supplementary file 4 — Supplementary Material 4 [file 13018_2024_4820_MOESM4_ESM.docx]

**Appendix 1:**

**1. Specific ankle provocation tests**

**a.) Dorsal flexion with weight bearing**

Step 1:


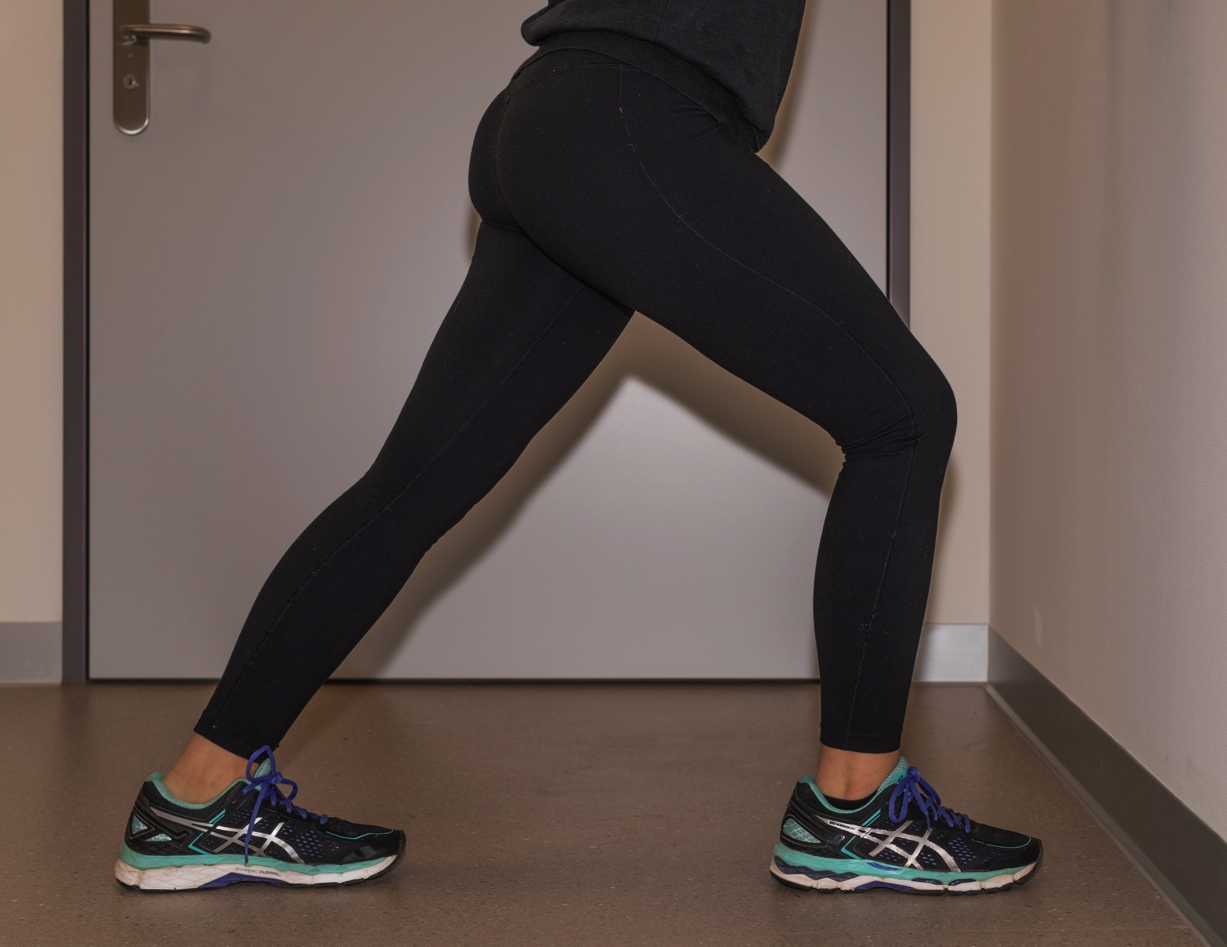


Step 2:


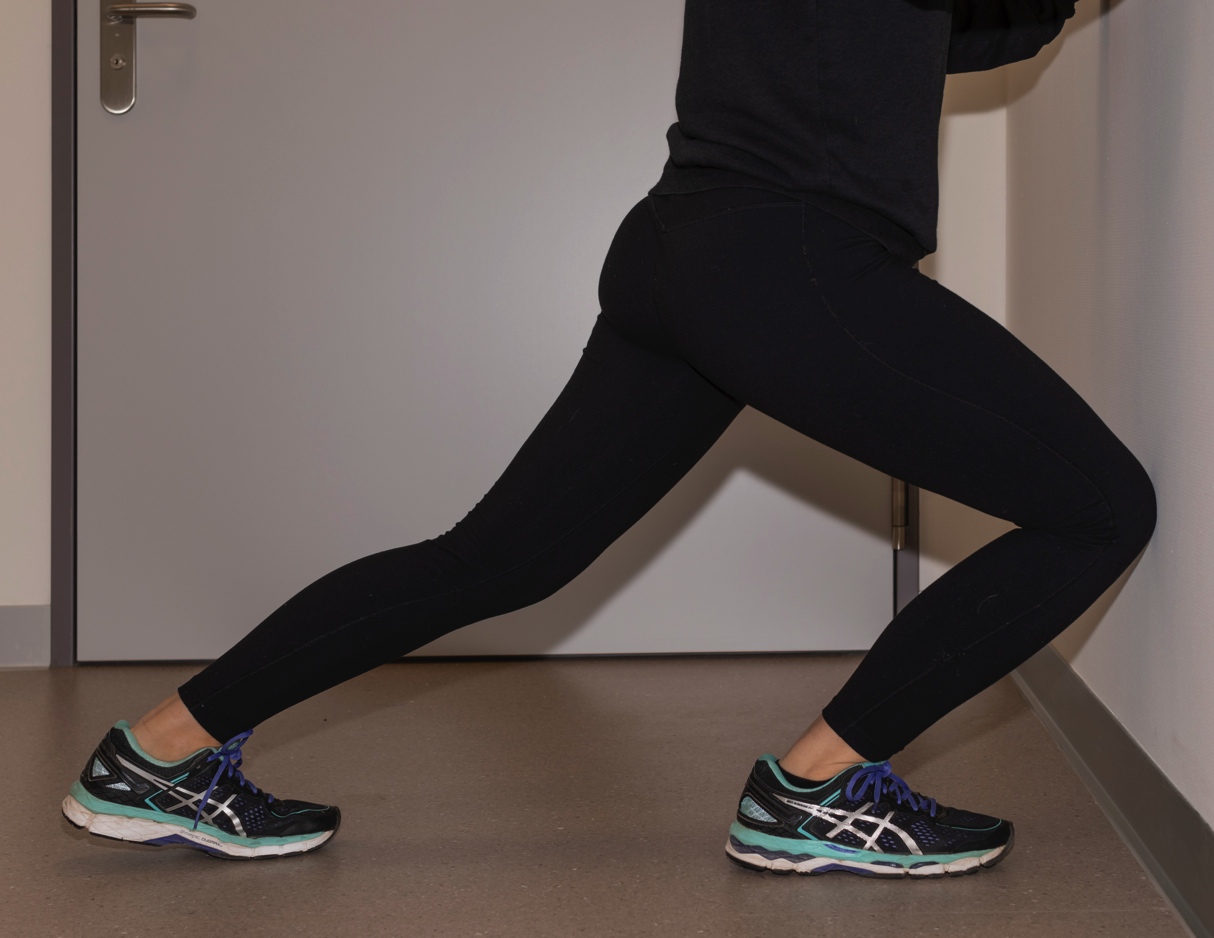


**b.) Y-balance-test**

Step 1


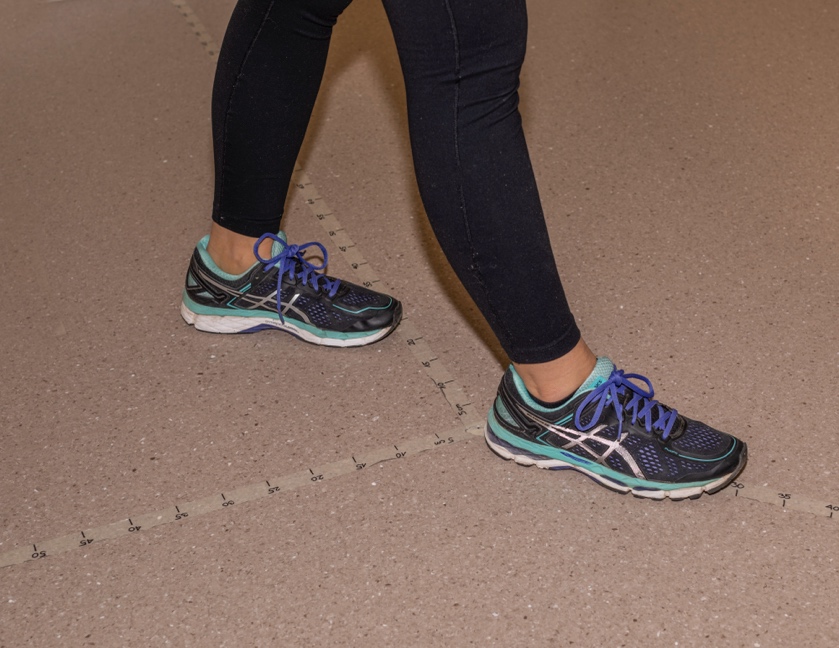

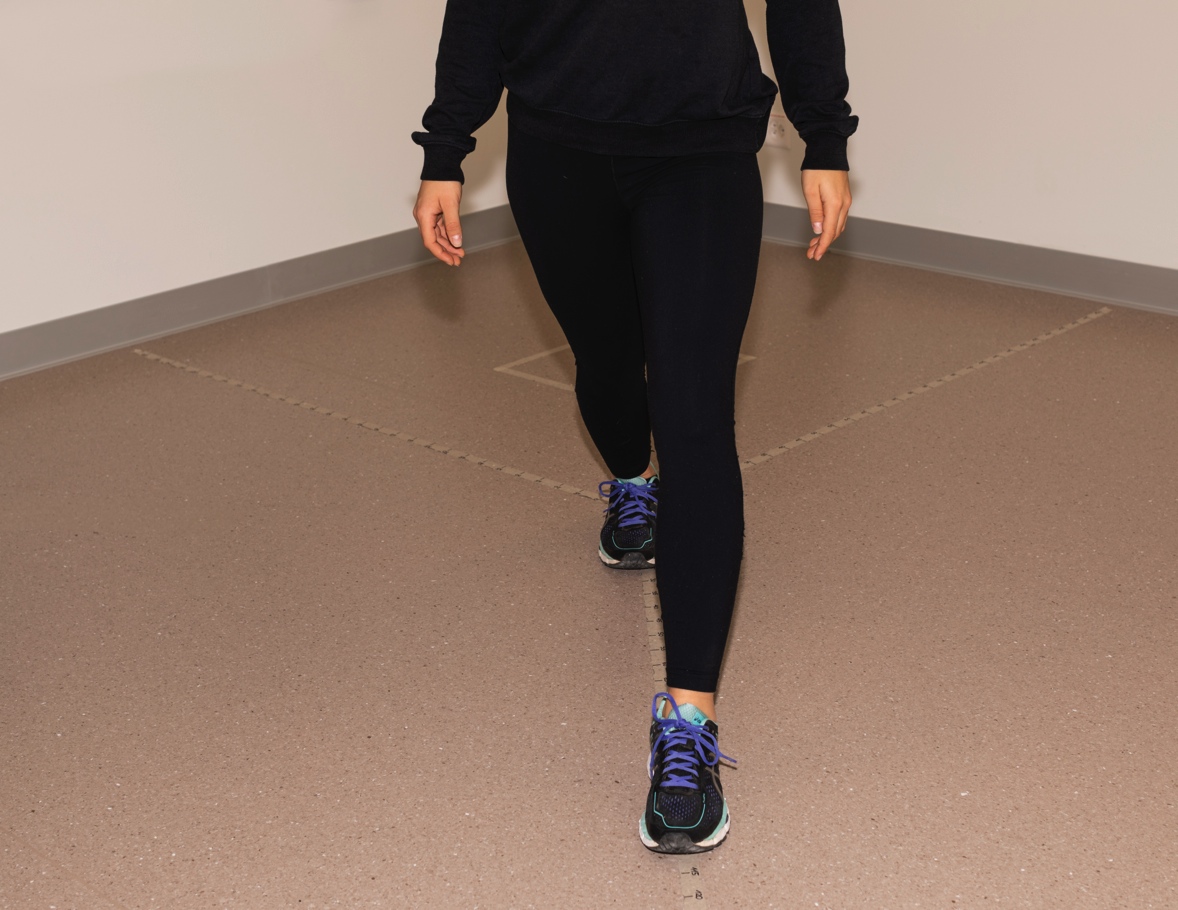


Step 2


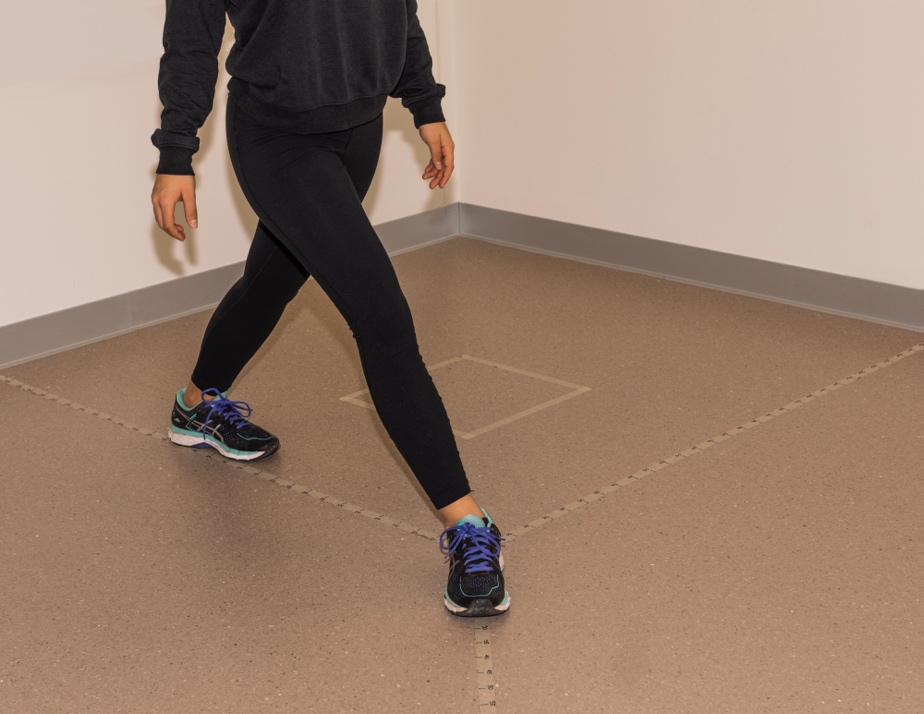


Step 3


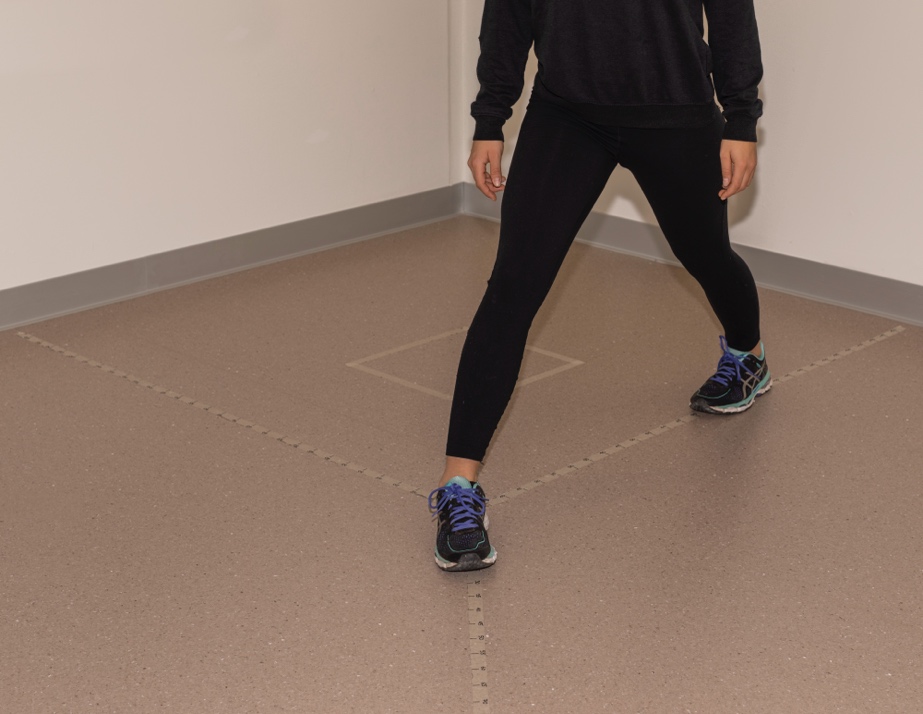


**c.) Modified drop-jump-test**

Step 1


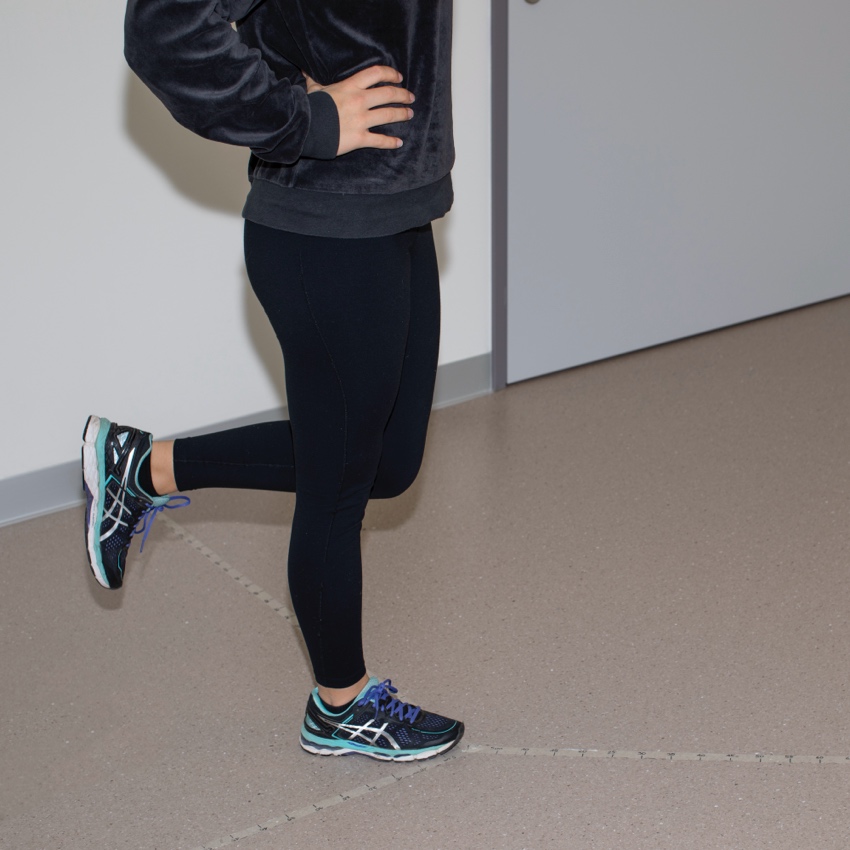


Step 2


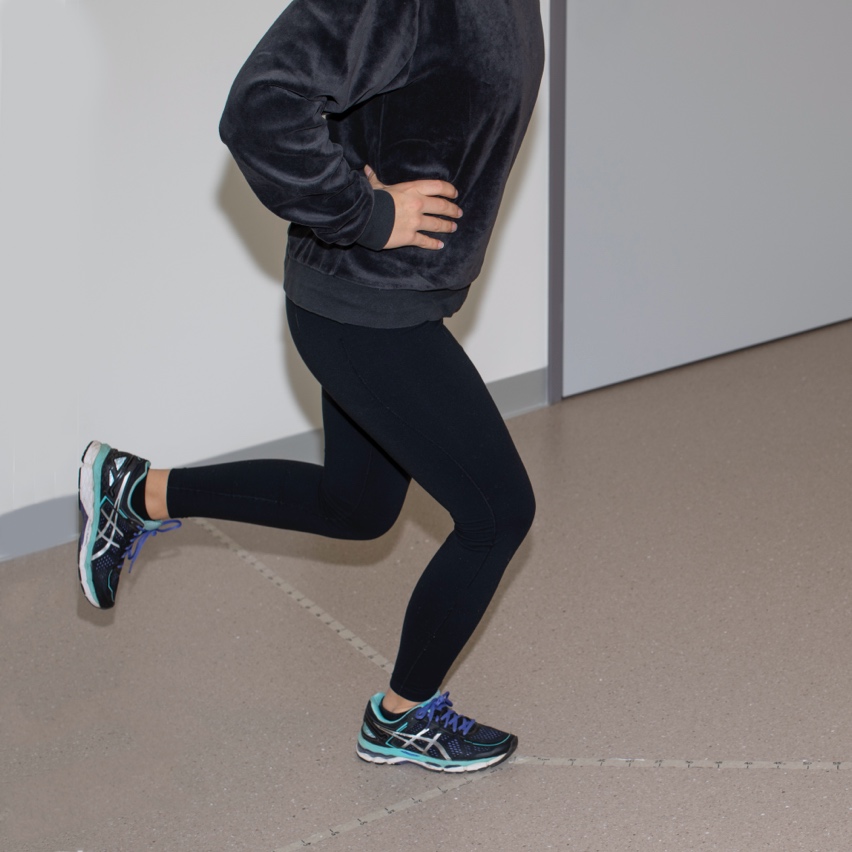


Step 3


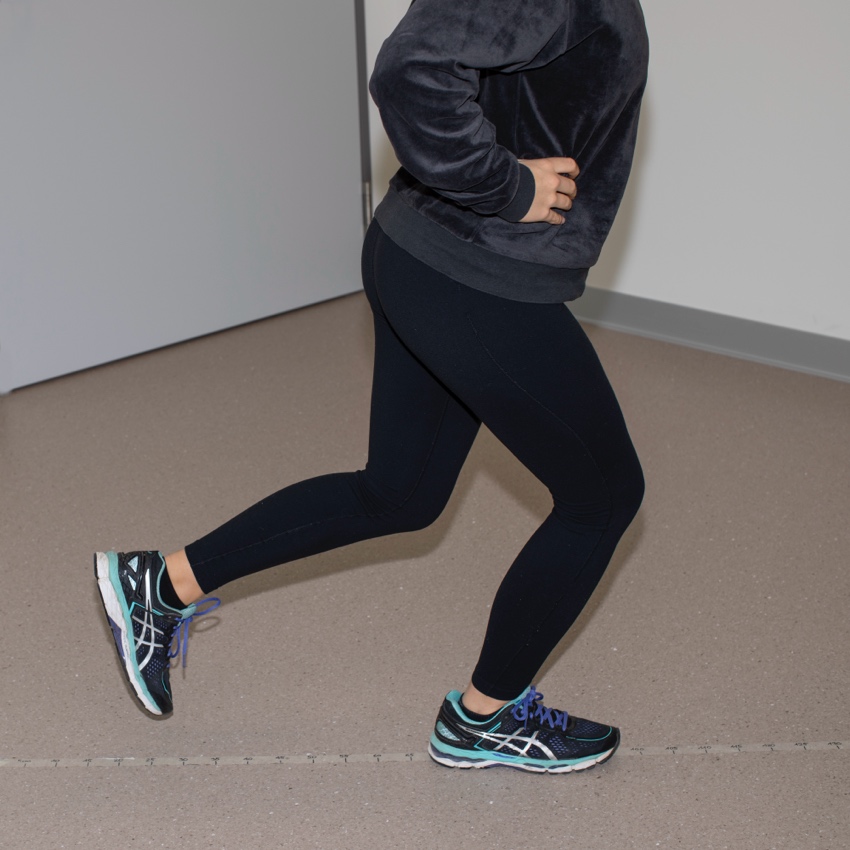


**d.) Modified leg-press-test**

Step 1


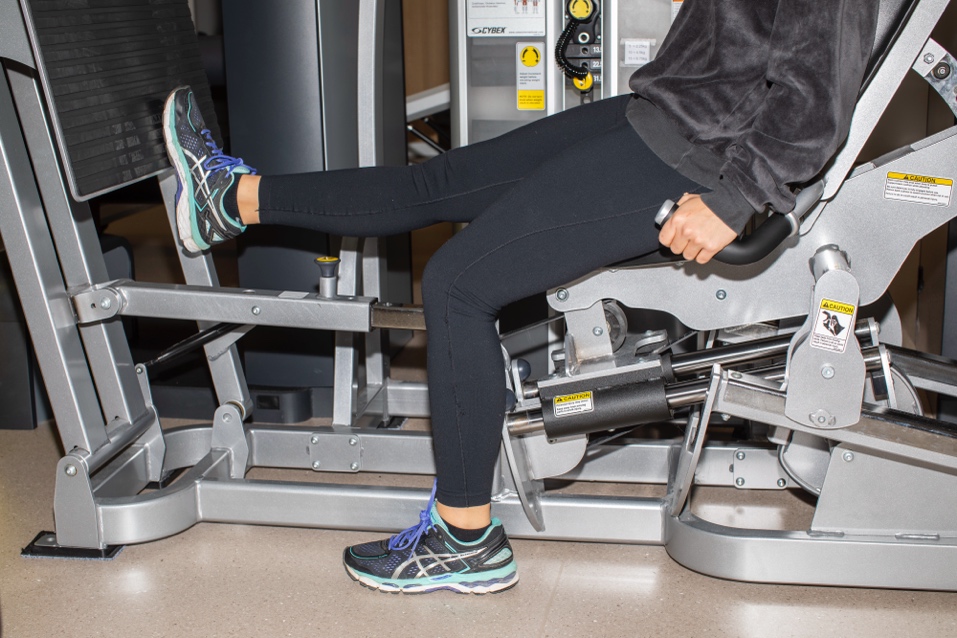


Step 2


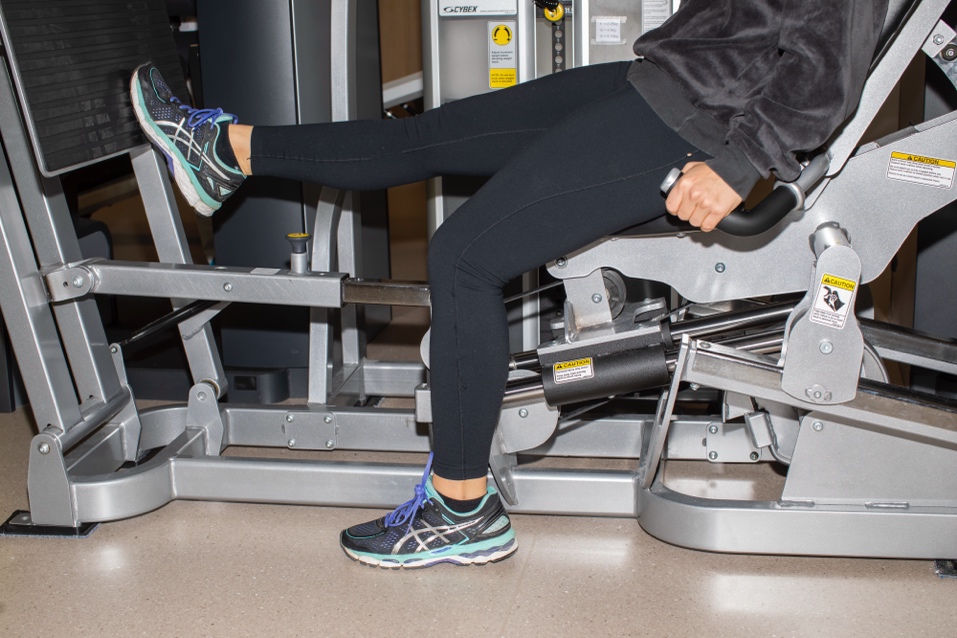


**e.) Maximum distance floor-to-heel-test**

Step 1

**
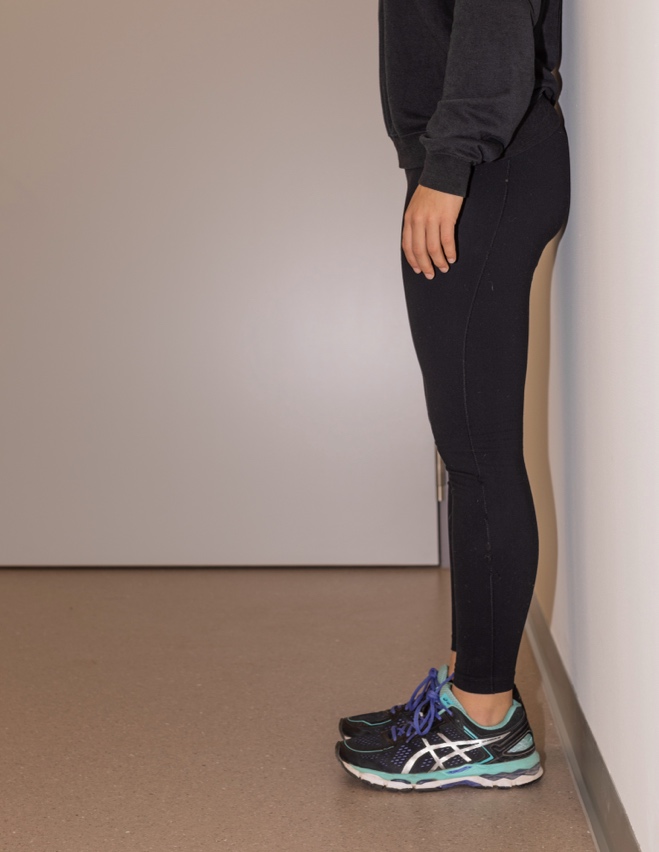
**

Step 2

**
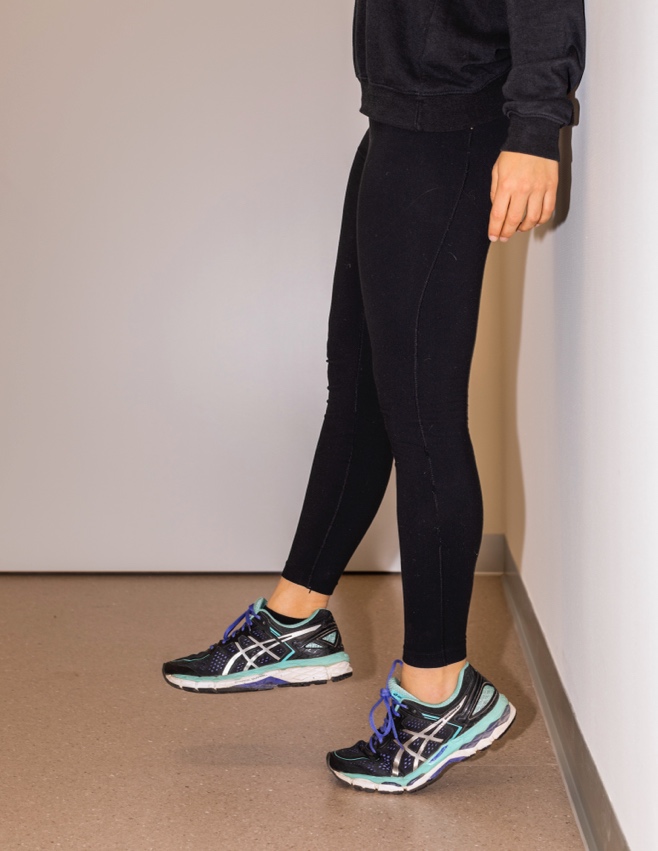
**

**2. Radiological evaluation**

**
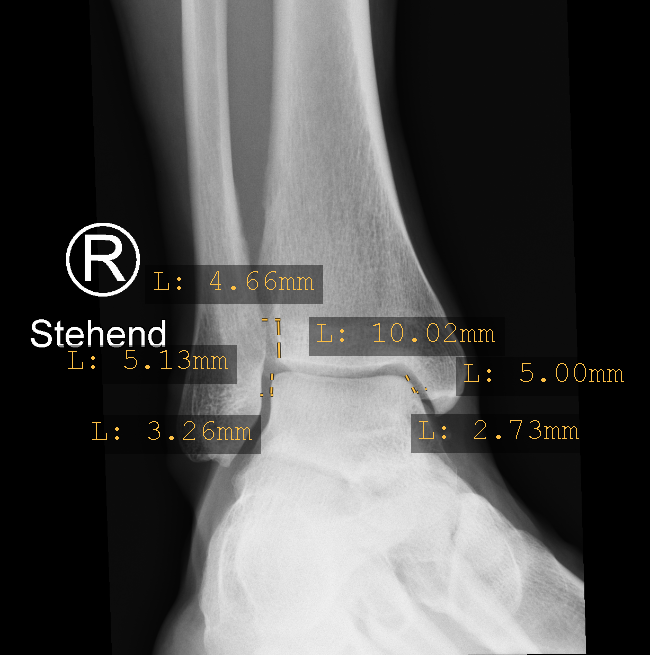
**

**X-ray of a right ankle in weightbearing mortise view (modified a.p.).** Red: Lateral clear space measured by the distance between the medial border of the lateral malleolus and the lateral border of the talar dome 5 mm inferior to the superior talar joint line. Blue: Medial clear space measured by the distance from the lateral border of the medial malleolus and the medial border of the talar dome 5 mm inferior to the superior talar joint line. Green: Tibio-fibular clear space measured from the point 1 cm above the lateral edge of the inferior tibial joint line (anterior ridge of the tibia) to the corresponding horizontal point at the fibula.


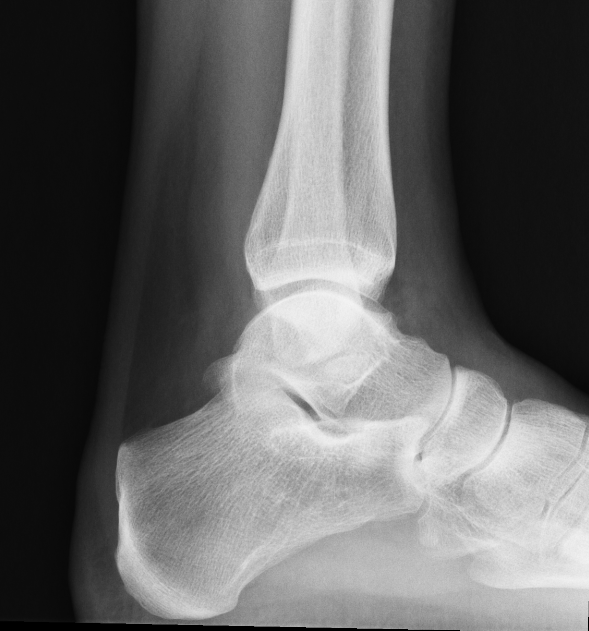


**X-ray of a right ankle in weightbearing lateral view with no signs of arthritic changes.**
